# The Challenges of Urban Ageing: Making Cities Age-Friendly in Europe

**DOI:** 10.3390/ijerph15112473

**Published:** 2018-11-05

**Authors:** Joost van Hoof, Jan K. Kazak, Jolanta M. Perek-Białas, Sebastiaan T. M. Peek

**Affiliations:** 1 Faculty of Social Work & Education, The Hague University of Applied Sciences, Johanna Westerdijkplein 75, 2521 EN Den Haag, The Netherlands; 2 Department of Spatial Economy, Faculty of Environmental Engineering and Geodesy, Wrocław University of Environmental and Life Sciences, ul. Grunwaldzka 55, 50-357 Wrocław, Poland; jan.kazak@upwr.edu.pl; 3 Institute of Sociology, Jagiellonian University, ul. Grodzka 52, 31-004 Kraków, Poland; jolanta.perek-bialas@uj.edu.pl; 4 School of Social and Behavioral Sciences, Department of Tranzo, Tilburg University, Professor Cobbenhagenlaan 125, 5037 DB Tilburg, The Netherlands; s.t.m.peek@uvt.nl

**Keywords:** older adults, older people, cities, housing, technology, dementia-friendly, Poland, The Netherlands

## Abstract

Urban ageing is an emerging domain that deals with the population of older people living in cities. The ageing of society is a positive yet challenging phenomenon, as population ageing and urbanisation are the culmination of successful human development. One could argue whether the city environment is an ideal place for people to grow old and live at an old age compared to rural areas. This viewpoint article explores and describes the challenges that are encountered when making cities age-friendly in Europe. Such challenges include the creation of inclusive neighbourhoods and the implementation of technology for ageing-in-place. Examples from projects in two age-friendly cities in The Netherlands (The Hague) and Poland (Cracow) are shown to illustrate the potential of making cities more tuned to the needs of older people and identify important challenges for the next couple of years. Overall, the global ageing of urban populations calls for more age-friendly approaches to be implemented in our cities. It is a challenge to prepare for these developments in such a way that both current and future generations of older people can benefit from age-friendly strategies.

## 1. Introduction

Urban ageing is an emerging domain in social and health sciences, with implications that reach far beyond the borders of these disciplines [1]. It deals with both ageing of the population and living in cities. One of the great achievements of modern society is the ever-increasing life expectancy of the general population. In Europe and the Western World as a whole, people live longer and generally in better health than before. The ageing of society is a positive yet challenging phenomenon, as population ageing and urbanisation are the culmination of successful human development [2]. Their interaction raises issues for all types of communities in various domains of urban living [3]. All over the world, there is an increase in the number of older people, including Africa, though this continent remains relatively young compared to other parts of the world [4]. According to the Organisation for Economic Co-operation and Development (OECD) [5], the population share of those of 65 years old and over is expected to climb to 25.1% in 2050 in its member states. Cities in particular have large numbers of older inhabitants and are home to 43.2% of this older population. In the context of urban ageing, cities are defined as an inhabited place of greater size, population, or importance than a town or village.

Understanding the relationship between population ageing and urban change, and the need to develop supportive urban communities are major issues for public policy [3]. Plouffe and Kalache [2] see older citizens as a precious resource. In order to tap the full potential that these people represent for continued human development [6], the world’s cities must ensure their inclusion and full access to urban spaces, structures, and services. Therefore, cities are called upon to complement the efforts of national and regional governments to address the consequences of the unprecedented demographic shift [5]. An important question in relation to urban ageing is what exactly makes a city age-friendly [2,7,8,9,10,11,12]? Another relevant question is which factors allow some older people in cities to thrive, while others find it hard to cope with the struggles of daily life?

This viewpoint article explores and describes the challenges to make cities age-friendly in the Europe, for instance, on the neighbourhood/community level and in relation to technology. Examples from projects in age-friendly cities in The Netherlands and Poland are shown and discussed in order to illustrate the potential of making cities more tuned to the needs of older people and the challenges encountered. The rationale for these two countries is that first of all, Poland is yet one of the youngest countries in Europe, but will soon be experiencing a rapid population ageing. Poland will see a sharp increase in the number of people aged 85 years and over, and will become one of the oldest populations in Europe [13]. Hence, policies focused on ageing population are relatively new [14]. The Netherlands has a long-standing reputation for age-friendly measures, innovative housing concepts for older people, and long-term care policies, and focuses on the active participation and vitality of the senior population [1,15,16,17].

## 2. The City as an Ideal Place for Older People?

Ageing in place (i.e., living in the community, with some level of independence, rather than in residential care), is often seen as an ideal [18,19]. However, there are numerous challenges concerning the adequate provision of services, safety concerns of older people, and affordability issues, which have led some researchers to argue that the focus should be on ageing in the right place [20,21]. Given the many challenges, one could ask the question whether urban environments are the best for an ageing population and, therefore, the right place to age well?

The OECD [5] report concludes that ageing trends are different between metropolitan areas and non-metropolitan areas. In the large urban areas, the older population is growing faster than the total population. This means that the challenges are greater to overcome, but then again, cities have more and better resources and offer greater opportunities. Smedley [22] once posed the question if “*for an ageing society to function there needs to be a movement back to the cities—but cities need to be adapted and designed with this in mind. Cities don’t always seem the most old-age friendly of places.*”

This leads to the question whether there is much of a difference in the challenges encountered by older people when ageing in urban versus rural environments [23]? Many researchers have studied the differences between urban and rural communities without a specific reference to ageing [24,25,26]. For instance, they found that at low levels of economic development urban areas are favoured over rural areas in income, education, and occupational structure. There seemed to be a larger life satisfaction among city dwellers. At more advanced development levels, rural areas approach or exceed urban in life satisfaction within the European Union [24,25]. In the United States of America, there is a gradient of subjective well-being (happiness) that rises from its lowest levels in large central cities to its highest levels on the small-town/rural periphery [26]. When it comes to ageing, the higher density of urban settlement ensures a closer proximity to public services, which influence the quality of life of older people. Due to the economic contexts, such conditions often cannot be provided in rural areas [23]. However, it should be highlighted that over time the living conditions are improving in non-urban areas, in particular in suburban zones in Europe [27,28], but likely the density of services will never reach that of city centres. However, an analysis of the rural and urban locations suggested that the living conditions are much better for older people living in cities than for those living at the countryside even the differences are not large [29].

There is increasing recognition that planning for later life is not about interpreting ageing in place as ‘staying put’ during the life course. Instead, according to Phillips [30], urban planning is now focusing on so-called active ageing [31], and older adults are seen as consumers and in various forms of employment. In relation to urban ageing, there should be a focus on promoting mobility within cities (such as walkability, use of public transport), promoting safety and security, and empowering older people in local communities [32]. Additionally, urban planning should stay away from the segregation of older people as exemplified in urban entities such as Sun City Arizona and The Villages of Florida in the United States of America that are directed specifically towards the needs of a single demographic segment [33,34]. A truly age-friendly city is not focused on just one generation, but includes and embraces all generations, which is also reflected in design principles including the universal design concept [35]. Ideally, places to live should be organised to facilitate social interaction and foster a sense of community [19,36]. Today, the creation of age-friendly cities is a world-wide movement [37,38,39,40,41,42,43,44], but there are regional differences in the approaches applied to creating age-friendly cities based on cultural contexts and financial conditions. Cities may be the best possible environment for older people to live and age in place, if they are under a cycle of continuous reinvention and adaptation to guarantee they are in line with the needs of an older population. This requires continuous efforts from various professional stakeholders in the domains of healthcare, social work, real estate, and governments.

## 3. Shaping Age-Friendly Cities

**Figure 1:**
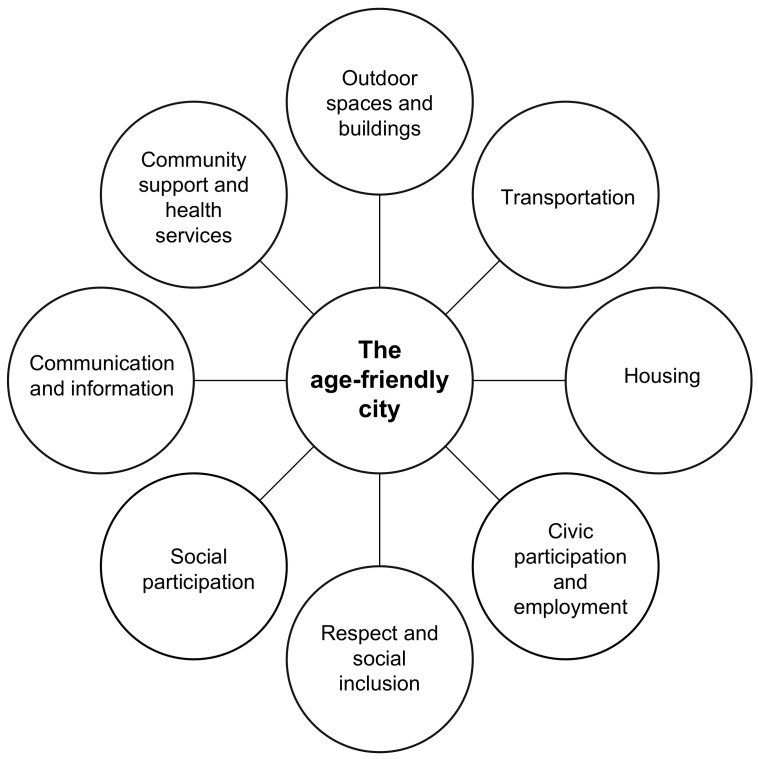
The eight domains of an age-friendly city [45].

According to Fitzgerald and Caro [34], an age-friendly city offers a supportive environment that enables residents to grow older actively within their families, neighbourhoods, and civil society and offers extensive opportunities for their participation in the community. In other words: a place where older people are actively involved, valued, and supported with infrastructure and services that effectively accommodate their needs. Plouffe and Kalache [2] described the efforts of the World Health Organization (WHO) to engage and assist cities in becoming more “age-friendly”, through the Global Age-Friendly Cities Guide and a companion “Checklist of Essential Features of Age-Friendly Cities” (Figure 1) [45]. An age-friendly city should ideally be inclusive and offer opportunities for all people living in the city, and not just older adults. The concept itself is rooted in Lawton and Nahemow’s ecological model [46]. This model articulates the dynamic interplay between individual adaptation and environmental alteration in order to maintain optimal functioning in older age. The WHO project proposed that an “age-friendly” city is one that promotes active aging [45]. Such a city optimizes opportunities for health, participation, and security in order to enhance quality of life as people age [2].

For the WHO project, numerous partners from 35 cities from around the world collaborated, for instance, through conducting large-scale focus group sessions with various groups of stakeholders [45]. Based on this research, the features of age-friendly cities were determined in eight domains of urban life. These domains are: outdoor spaces and buildings; transportation; housing; social participation; respect and social inclusion; civic participation and employment; communication and information; and community support and health services. One of the noteworthy aspects of this global study was that there were no systematic differences in focus group themes between cities in developed and developing countries, although the positive, age-friendly features were more commonly seen in cities in developed countries. 

Despite the fact that the age-friendly cities initiative includes cities from all over the world, there is some critique that is may be Western-oriented and could have missed some of the age-friendly aspects that are critical for older adults’ everyday life in cities in a developing country [47]. One of the major world cities that have adopted the principles of age-friendly cities is Hong Kong. The Hong Kong Special Administrative Region Government [43] stimulates active and healthy ageing by focusing on a multi-dimensional approach. The dimensions include financial adequacy, general and hospital care, community and residential care, transport and mobility, housing and the built environment, active ageing, more flexible employment, and family-friendly measures. These family-friendly measures reflect the view on age-friendliness in the Eastern context. The local government takes actions in all these domains to make cities more inclusive for the older population and for others as well. Typical challenges in Hong Kong are the provision of home modifications in small apartments in high-rise buildings, and creating accessible modes of transportation in the city where the vast majority of people rely on public transportation. The Hong Kong Special Administrative Region is just one of the examples of local governments taking place-specific actions in the field of urban ageing.

According to the OECD [5], ageing societies pose diverse challenges, such as redesigning infrastructure and urban development patterns, social isolation, lack of accessibility and housing affordability. The OECD also mentioned a large set of opportunities that society can benefit from, not just enterprises and professionals, but also older people themselves who can benefit from and contribute to ageing well in the city [48,49]. These opportunities include new developments in technology and innovation, market approaches to retrofit existing housing facilities that allow older people to maintain or regain their autonomy, and the organisation of services for older people by older people in voluntary networks. In addition, there is room for the development of the so-called “silver economy”, especially with the development of a white sector, i.e., all professions related to the medical and healthcare sectors [50]. And many of these opportunities usually require support from policies, which means that local policy makers are increasingly engaging in work related to the ageing of society in the broadest sense of the term, instead of just care support and pension schemes which is at country level implemented and/or changed. Policies for ageing societies should be a way to prepare for the future [51]. Of particular interest are the built environment and the use of technology.

## 4. Building Friendly Places and Inclusive Neighbourhoods for an Ageing Population

There are many questions that need to be addressed when building friendly places and inclusive neighbourhoods. For instance, what does it mean for the built environment and urban planning when a city’s population is ageing? How does population ageing and older people’s housing needs relate to a growing demand for real estate by younger people wanting to start a professional career in the city after graduating from university? How can related services meet the needs of the ever-diversifying urban population? And, who are the stakeholders responsible for providing these services, especially in times of shortages in the number of professional carers?

An important issue is affordability. Large and economically-growing cities are known for high real estate prices and a high demand for residential space from both the old and the young, and in some cases, from international investors and the informal tourism sector. This may imply that designer needs to come up with small dwellings that are more affordable. Such types of housing may also meet the needs for older people who are less affluent and have difficulty paying the rent or find it hard to obtain a mortgage as they no longer actively participate in a work life after retirement. Homes that become vacant after moving, in turn, become available on the real estate market for others to buy or let. In essence, the lack of space and limited financial means are not the main drivers for small dwellings. In the Western World as a whole, it is the demographic fact that the growing number of older people are, in fact, single-person households, made up of people who have been single all their lives (without having any offspring), or who divorced or became widowed in later life. In various studies (for example, Onolemhemhen [52]), it was found that among older people, there is a larger percentage of poor women than men [53]. Nevertheless, both groups are at risk of dropping out of society (social exclusion), despite numerous personal and environmental strengths, particularly when the costs of housing take up a disproportionate share of the living allowances. When people are facing increasing costs for health care services and medication, or the rising costs of energy, financial challenges emerge and older people are at risk.

There are other developments that call for a different approach to the size of housing for older people. Instead of building smaller homes and apartments in cities to accommodate our ageing populations, a new sense of community could lead to a demand for larger dwellings, which could be dwelled by multiple tenants at the same time. This kind of group living encompasses living with like-minded people, with friends or old acquaintances who share similar interests. In such group living arrangements, co-optation methods could be applied to select suitable co-residents. Having bonds with co-residents and other people, and to be able to engage in meaningful activities, is known to contribute to a sense of home among nursing home residents [54,55], and it is probably also true for people living in the community. The social housing associations, commercial project developers and private investors can help develop such group-living spaces. There are lots of opportunities for people who want to live together in terms of shared resources, cooking and eating together, keeping an eye and helping a co-resident when he or she falls ill. Again, people who have found themselves divorced or widowed, and feel lonely or left-out, may find it attractive to start living together. The same may be true for a group of people who have always been single but miss the interaction with others that they used to have when still employed or active in organisations. 

For many people living together with like-minded people seems to be an ideal. In multicultural urban environments we have witnessed the emergence of nursing homes and housing for older people with a comparable cultural, ethnic, social or religious background. Many of the large cities in the Western World have a multicultural and multi-ethnic build-up of their societies [56], and each of the groups have their own needs and preferences in terms of housing and interaction with each other. The Netherlands has housing for groups of older people with roots in the colonial past, for instance, people with a Netherlands East Indies (and/or Indonesian) and Surinamese background. In addition, there are group living arrangements for older people with Turkish, Chinese and Moroccan backgrounds, in which housing and social relationships are tuned to cultural and even religious needs. Foods served in these houses reflect the rich cuisines from the former homelands. Despite the discussions on whether such buildings and communities are or are not an example of segregation in our societies, they do serve a role in getting people to live with like-minded others. 

The built environment can contribute to meeting others, even at old age, as well as social engagement with like-minded people. Community building is about stimulating the sense of belonging and sense of community among older people, and between the generations. The importance of it was also noted by Rémillard-Boilard et al. [57], who called for the promotion of social connectedness within urban environments. This means creating opportunities for meeting others, also outside of your own familiar age or socio-economic group. Cities are important sites for building social networks but can also trigger marginalisation and social exclusion [58,59], due to the individualised lifestyles of the people. There are many ways for practitioners to promote social engagement. Ideally, multi-generational and non-ageist approaches [60] are employed to create truly inclusive and age-friendly cities with a special focus on ageing as the urban vulnerability factor [61].

Building friendly places and inclusive neighbourhoods is also about modifying existing homes, designing homes that are fully adapted to older people, and about creating accessible neighbourhoods with an adequate provision of services [18,62]. Easy-access or level-access and single-level dwellings are among the types of homes needed to house the growing group of older people. Growing older goes together with an increased risk of reduced mobility, and older people may also be prone to imbalances or falls. Measures to improve the accessibility of homes may also be beneficial for the younger generations, including young parents with prams, or younger people with physical limitations. When considering the accessibility of homes, one should also discuss the concept of egressability. In other words: are people able to leave a building in case of calamities? Such events include fires or being taken away on a stretcher by an ambulance worker, or in extreme and terminal cases, in a coffin upon death. Many buildings provide insufficient opportunities for older people and people with mobility impairments to be evacuated smoothly, as elevators cannot be used in case of fires or because a stretcher is too large to turn around in a corner in a narrow corridor.

Other mobility issues are encountered in public transport with the use of accessible busses that take people around town, sheltered and clean outdoor seats for people to take a rest, sufficient, clean and accessible public toilets, and even adjusted level-access sidewalks that are accessible for people using wheelchairs and wheeled walkers (and again, younger people with prams). All these elements of urban design have an impact on the so-called walkability of neighbourhoods. Access to public services, better commutes and proximity to other people and places make neighbourhoods happier, healthier and more sustainable. Neighbourhood walkability is not a new approach in academic research as a measurement of promoting active urban ageing [63,64,65]. There is a need to undertake actions in improving urban walkability conditions, as they are strongly related with the quality of life of (older) citizens [66]. Urban planning challenges include an even and accessible distribution of services, including shops and health centres, which do not require large distances to travel. 

## 5. Technology as a Solution for Urban Ageing?

Because of exponential technological advances in the last decades, the use of smart technology is increasingly looked at as a possible solution for dealing with the some of the challenges related to urban ageing. Urban populations can be the front runners in the use of technology. Calvert et al. [67] found that technology use is common by very old community-dwelling older adults. In the study, urban respondents were more likely than rural ones to use consumer electronics including computers. Righi et al. [68] provided a vision of smart city, which conceives of older people as embedded in intergenerational urban communities and capable of creating new engagement situations by reconfiguring IT-driven scenarios to their interests and social practices. Looking in more detail at the concept of smart cities, many definitions of a smart city exist, none of which has been universally acknowledged [69]. The concept may be understood as “urban areas that widely utilise information and communication technologies (ICTs) to organise and provide all urban functions, for instance, to reduce costs of infrastructure maintenance (such as roads, bridges, subways, airports, seaports, public transport, and sewerage), consumption of resources (such as gas, electricity, and water supply), better use the free spaces as well as to engage citizens in local governance” [70,71]. Specific examples related to the aging population include health monitoring and emergency response systems, wandering detection technology, and the automated assessment of the need for assistance in activities of daily living [72,73]. Smart city technologies rely heavily on both Big Data analytics and the Internet of Things, which includes the diffusion of sensors and wireless sensor networks in the city with the capability of real-time data gathering [74]. Such real-time data gathering can also be accomplished inside older adults’ dwellings, effectively turning these into so called smart homes. Smart homes have been postulated as a potential solution to support ageing in place. For example, smart homes technologies are aimed at supporting independent living by facilitating tasks such as preparing food and cleaning. Furthermore, smart home technology can assist in monitoring and maintaining health status [75]. Despite the emphasis on smart homes by government agencies, policy makers, and the industry, their existence is not widespread [76,77]. Consequently, their suggested potential for older adults for alleviating pressure on (family) carers, and decreasing health care expenditure, has not yet reached its full potential. One of the reasons for this is low level of smart home technology adoption by older adults [78,79].

When seeking to understand technology acceptance by older adult who are ageing in place it is important to acknowledge that the older adult population is highly heterogeneous [80,81]. Older adults do not only vary with regards to their values, attitudes, needs and wants, but also with regards to how these are affected by ageing, life events, and changes in their social and physical environment [82]. These differences are also reflected in their use of technologies that could help them to age in place [83]. Whether or not a new technology is considered a welcome addition by a senior is dependent on perceived benefits and costs of technology, perceived need for technology, social influences, and the degree to which a technology is in line with the older adult’s self-concept [75,84,85,86,87]. Furthermore, that the use of technology is dependent on the availability and use of technological and non-technological alternatives [79,88]. For example, older adults who have family members that visit them daily are less interested in smart home monitoring technologies that are designed to watch over them; they see no need for it.

As long as there is technological development, there will likely exist a gap between those that grew up with certain technologies, and those that did not [89,90]. Consequently, older adult can benefit from people around them who can help them encounter technologies, and who can also help them in using technologies. For seniors, assessing what is the most appropriate technology for their ageing in place needs can be difficult. Professionals (i.e., technology consultants) specifically tasked with matching seniors’ needs with technology solutions can greatly help here. In the municipality of The Hague, a participatory action research project was conducted to determine the challenges these professionals face and to co-design tool for optimising their matchmaking service. Results showed that important challenges for technology consultants in their current matchmaking practice were: making the matchmaking service more demand oriented and creating an accurate and complete overview of relevant factors within the seniors’ individual situation so that an optimal match could be made. A matchmaking tool was created to help overcome these challenges. The tool entails a structured approach to better match technologies to a senior’s individual ageing in place needs and circumstances [91].

## 6. Age-Friendly Cities: Examples from the Netherlands and Poland

There are numerous cities in The Netherlands and Poland, which are either part of the WHO Age-Friendly Cities consortium or which implement strategies for age-friendliness without being a consortium member. In Table 1, examples from the city of The Hague (*Den Haag or ’s-Gravenhage*) in The Netherlands and the city of Cracow (*Kraków*) in Poland are described in relation to the eight domains of age-friendly cities. This overview reflects the spectrum of projects for older people that both cities support not aiming to present a complete view/picture. Data are based on the most recent statistics available for these two cities. These examples are followed by a number of challenges that are identified as relevant for the coming years.

In 2014, there were 70,200 people in The Hague aged 65 years and over, and 31,100 were aged 75 years and over [92] out of a population of just over 500,000. The percentage of older people (65+) in the city was thus 13.8%. Older people with a migrant background were becoming more numerous: in 2014, and a total of 28% of the older people had such a migrant background, a figure expected to rise to 32% by the year 2020. Over three quarters of the community-dwelling older people live in a multi-storey building. About 40% of older people (65+) live in a home that is labelled as a home for older people, a nursing home or a so-called life-time home, of which a large section is made up of social housing. Of all senior household in the city, about 17% have to live of a minimum income (often a state pension). About 61% of the older people are able to use the Internet [92]. About 60% of older people have a physical limitation or chronic disease; 30% of older people deal with a limitation in daily functioning, and about 10% deals with the effects of dementia syndrome.

Data for Cracow show that the city has about 760,000 inhabitants (2017), with more than 21% of people aged 60 years and over. The life expectancy at birth for women is 83.1 years, while for men it 77.1 years, and the values are among the highest in the country. The share of older persons in the city of Cracow increases in the years to come, while the youngest age group (0–17 years) decreases and it will only be about 16% in the year 2030. Cracow is attracting expats and foreigners to work in the city but exact numbers are difficult to present. In general, older Polish citizens live in their own flats and single-family homes. Due to lack of sufficient adequate institutional settings like nursing homes only those, who are very dependent and without relatives, are eligible for admission to these facilities. Others can apply depending on the available places. The exact numbers on how many Internet users aged 60 years and over live in the city of Cracow are again not easily available. Concerning the health status of older people in Cracow, the majority have a physical limitation and/or chronic diseases [93].

The examples of The Hague and Cracow show the variety of strategies and projects seen in age-friendly cities in both countries, and the level of maturity of the implementation of these initiatives. Overall, the examples show how differently and similar the city could be age-friendly taking into account not only the financial aspects (limitations and opportunities of the public funds), the organisational contexts (infrastructure, including technological options), human resources (people who are trained to change the city into an age-friendly place, and who are willing to contribute thanks to the power of policy making) and cultural contexts (on both the national and city levels ideas and project may be well or poorly received). In this way, we try to show that examples presented could be found in many European cities (see [96,97,98,99]). Through sharing the best practices among policy makers, other places try to implement similar solutions in their own cities, in a way that fits the local conditions and contexts. 

Many of the examples from The Hague and Cracow have not been properly evaluated in order to understand if the efforts to create age-friendly communities are turning the curve to create healthier, more supportive and more inclusive environments for older adults. The overview of projects in both The Netherlands and Poland offers ideas for programme and policy implementation in age-friendly communities, but evidence that these interventions demonstrated measurable change in various outcome domains, such as quality of life, social participation, financial stability, technology literacy and so on, is lacking. There is a need to gather and systematically evaluate outcomes or impacts measured over time of all age-friendly programmes and projects, in other to make a more powerful case about the need and usefulness of such initiatives. Still, some preliminary statements about the efficacy of such initiatives can be drawn. Similarly, since so many age friendly communities have various community leaders and entities who assume responsibility for the widespread education and advocacy of age-friendly improvements, it would be helpful if there was a clear understanding of who successfully implemented all age-friendly interventions and what resources were required for their successful implementation. In the end, the availability of specific information such as a measurable impact, the professionals involved, financial aspects, and resources required for the implementation, could provide more meaningful guidance for professional practice in age-friendly cities. 

The three main challenges for the city of The Hague [92] are (1) the improvement of the vitality of its older citizens, (2) combatting loneliness and focus on satisfaction in life, and (3) ageing-in-place. When the vitality of the citizens is improved, it is believed to improve feelings of loneliness (52% of older people in the city experience these feelings), particularly when losing a spouse and when the social network becomes smaller. The more vital older people, the more they are expected to take care of things themselves and to participate in society. This, in turn, should also lessen the demand for care. Setting goals in life should be in accordance with one’s health status and living conditions. Ageing-in-place is stimulated through a dedicated availability of formal and informal care, and sufficient support for family carers. In addition, safety and security in the neighbourhood, meeting others and mobility are stimulated and supported. In addition, the municipality seeks attention for safety, security and domestic violence and elder abuse. For older people with dementia, a strong network of services in the neighbourhoods for support, counselling, instructions and day-care is set up for both people with dementia and their informal carers. Combining the potential of other sectors, including the arts, culture and sports with aged-care should challenges people to come up with new life goals based on their personal abilities.

In the case of Cracow, there are numerous challenges that were presented in the Programme of Social Activity of Senior Citizens [93]. Some of these challenges are somehow related to the challenges identified by the municipality of The Hague, which are as follows: (1) a need to support the education of older people; (2) increase opportunities for active and healthy ageing/lives; (3) increase the participation in social activities which promote inter- and intragenerational integration, and (4) support the social participation of older persons in general. Besides these challenges, it was noticed that loneliness (social isolation) and lack of ICT skills with a limited use of technology and the Internet among older citizens of the city are in an urgent need to be addressed by city policies and actions. The relative maturity of initiatives in The Hague and Cracow are shown in Table 2, which also shows the room for improvement in policy and practice.

## 7. Conclusions

The global ageing of the urban populations calls for more age-friendly approaches to be implemented in our cities. It is a challenge to prepare for these developments in such a way so that both current and future generations of older people can benefit from age-friendly strategies. This requires all public and many private partners to work together, for instance, in the redesign of the public space, healthcare and welfare services, and the design of new housing concepts and technologies. In order to achieve a truly age-friendly city, initiatives should be under a continuous cycle of evaluation and validation of the eight domains of the age-friendly city through the active involvement of older people who voice their opinions and experiences. The overview of projects from The Hague and Cracow showed that not all eight domains are evenly covered by projects, programmes and initiatives. This may be the result of the level of maturity of a city or country being age-friendly, or simply the result of policy choices made by governing bodies at the municipal level. Based on these data, cities can adjust to the future population profiles which will without a doubt be more senior than ever.

In this paper we referred to the well-known concept of age-friendly cities. We tried to shift the current perspectives towards the increased use of technology and towards being more open to and understanding of policy makers in their engaging in adequate actions to organise this environment as friendly as possible for all ages. The cases from both cities showed that there are many options to act at the local level with various initiatives and programmes, which make a real impact to the ageing population. Such actions, however, need constant improvement and adjustment to the needs and possibilities of the ageing population over time. Evidence from other cities -not only in Europe—which follow this path is building. This is an invitation to make a step forward in making age-friendly cities, not just in rhetoric, but also in practice, and to share best practices through easily available evidence and data.

## Figures and Tables

**Table 1:** Overview of age-friendly city priorities and projects in The Netherlands and Poland.

The Netherlands (The Hague)	Poland (Cracow)
**Social Participation**	
Older people are stimulated to participate in activities, which are affordable. Community centres are run in each neighbourhood. Restaurants in various neighbourhood are made accessible for older people and tuned to their needs. There is extensive collaboration between theatres, care organisations, sports organisations and the municipality in organising activities for seniors. The municipality established a knowledge centre for benefiting from peer knowledge and skills, set up meeting places for meeting with neighbours, and initiated programmes to fight loneliness. Some of the public parks have gym equipment for older people.	There are newly-established so-called Centers of Senior Activities in which persons over 60+ can meet on weekdays for various activities. The Centers need to be opened for at least 4 h per day, are financed from the city’s budget (seniors pay just about 1 € as a membership fee per month). Social participation is also possible via other formal and informal programmes, groups organised by non-governmental organisations, senior clubs, Universities of the Third Age etc. In order to promote the participation of older citizens, there are opportunities to visit museums, theatres, and concert halls for free or at a reduced rate.
Continues on next page **Communication and information**	
The municipality is generating familiarity with the use of modern media and the supply of services through these new media. There are practical solutions for older people who do not use the Internet (yet), including Q&A services for older people in community centres, for instance, supplied by students. Libraries offer courses in the use of computers and tablet computers to older people. All public information for older people is written understandably and, in an age-friendly font.	The communication between and for seniors in Cracow is taking place in various ways like: the government website [94], seminars, information points in locations of the Centers of Seniors Activity and a special issue once per week in local newspapers. There is also an increasing group of seniors using new communication technologies. One of the first free programmes in Poland for increasing ICT competences/skills for seniors has been running in the Regional Library in Cracow (The School of Active Senior with the Polish-German Association).
**Civic participation and employment**	
There is a system of reimbursement of costs when engaging in volunteering activities. The municipality has a stimulus package for increased chances for older people on the labour market. Since the 1980s, The Hague has a structure for the participation of older people in decision making on a municipal level named *Stedelijke Ouderen Commissie* or Urban Commission Older People. There are various municipal programmes for smart home technologies, and official support groups for informal carers to exchange experiences and get mental support. There are entrepreneurial programmes for older people set up in the municipality of The Hague. One of them is *Ouwe Koek*, which is a baking programme for older people. The baked products, including biscuits, are sold by volunteers and the money is used for leisurely activities and other programmes.	The great achievement in engaging the older persons in being active citizens was a possibility to establish at the city level so called Seniors’ Councils (*Gminne Rady Seniorów*) being voluntary, advisory and consultation group of older citizens which do not have a right to vote but to suggest to local policymakers/politicians what is the most urgent to solve. In Cracow there is such a body established, and together with others Councils it created an umbrella organisation called the Coalition of Seniors Councils, in order to be more representative at national level. Also, in Cracow the citizens can vote for the project which is then requested to be financed (*Budżety Obywatelskie*). Due to relatively lower eligible retirement age compared to other European countries, many Polish pensioners are not interested in employment only when they financial situation requires it. The exception is for well-educated older persons and while Cracow is a university city, it allows these persons to be additionally engaged in different forms of activity.
**Housing**	
The municipality is supporting the development of affordable life-time houses in partnership with social housing associations and project developers. There is a specific focus on the aspects of accessibility in the built environment. One of the main goals in the domain of housing is making people feel safe and secure at home. There are special housing programmes for older people with a LHBT background (*Foyer Coloré* initiative), or for people from an ethnic minority (such as *De Chinese Brug* for Chinese seniors or *Eekta* and *Rukun Budi Utama* for seniors from Suriname). These initiatives allow like-minded people to spend the latter days of their lives together.	The special housing programme for seniors is not yet widely developed in Poland, meaning affordable and at the desired quality of living conditions. Cracow as one of the oldest cities in the country, and heritage buildings were not destroyed during World War II. The existing old buildings, particularly in the old town’s centre, are not prepared for older persons (such as, no lifts, the cost of heating is high). Managing living conditions that answer to expectations and needs of the ageing population of the city proves challenging. There are private companies that aim to fill the niche and offer flats for seniors but due to the high cost it this is not yet a common form of housing in the city.
**Transportation**	
The municipality takes care of well-maintained bicycle lanes and pavements with even surfaces that allow water to run off after a shower. It also maintains a sufficient number of bicycle parking places in the city centre. Currently bicycle lanes are being broadened. There is free high-frequency public transport for older people, with senior-friendly staff (who received training) and stops close to home. There is a delivery service for medication by a pharmacist to the homes of older clients. There are voluntary taxi services for participation in church life or to go see a medical doctor. There are free rental services for mobility scooters.	In Polish cities such as Cracow the programme of free transportation has been popular for a long time already, such as for seniors aged 70 years or older in Cracow. It increases the mobility and transportation options for seniors who mostly use the public transport to get around in the city. There also exists a collaboration with a taxi company with a special reduced rate/discount if the ride is needed for a senior client (for example, to go see a physician).
**Community support and health services**	
The municipality actively supports social networks for informal care, which includes education and mental support. The municipality looks after an improved quality of home care services. The municipality stimulates the involvement of people aged 60–70 years and over in the provision of care provided to people aged 80 years and over. There is a small financial stimulus for informal carers of several k€. *De Boodschappenbegeleidingsdienst* (BBD) is a service that assists older people with doing groceries and helps prevent feelings of loneliness.	At the city level, the community support is crucial but besides the social assistance system financed by the city mostly for those with low income, there is more informal and family care practiced. Even neighbourhood relations involved in this type of services. In some local parishes, there are also voluntary groups supporting seniors. In addition, day care centres are needed to be further developed as current numbers are not enough. There is no local/city financial incentive for informal carers to support older adults. Even though there are unique projects that recently tested the use of ICT, telemedicine and telecare technology have not yet been explored enough.
**Outdoor spaces and buildings**	
There are well-maintained green spaces in the neighbourhood, which are cleaned and pruned on a regular basis in order to stimulate older people to go to parks. The municipality takes care of the availability of a sufficient number of outdoor benches, which are high enough and dry. In winter, there is a plan to prevent the accumulation of ice and frost on streets for safety. Mopeds are no longer allowed to use bicycle lanes for improved safety. There are safe and user-friendly ATMs. There are a number of accessible urban farms.	The buildings and the public institutions are more and more prepared for seniors and persons with limitations, as well for families with children to make them easily accessible. In cities there can be observed more and more efforts to create green zones/parks with benches and adequate infrastructure. The outdoor gyms are new in many places in cities, being open and for free for all citizens, regardless of age.
**Respect and social inclusion**	
There is a stimulus package for care and assistance provided by neighbours as informal carers, and this is supported through municipal policies. As the church is withdrawing from daily life, social activities are increasingly important, such as meeting groups, which are supported by the municipality. Schools can adopt a nursing home, and such actions focus on intergenerational interaction between pupils and older people.	The inclusion of older citizens through various activities via Senior Activity Centres (already more than 30 created in Cracow city from public funding, offering various courses), cities offer dancing events for free, outdoor activities (during spring/summer in parks), intergenerational activities (like doing jointly the theatre performances), singing etc.

Source: The projects from The Hague in The Netherlands are taken from van den Berg et al. [95]. The projects from Poland are taken from various sources. Unpublished materials from the web pages of the Cities of Cracow, a few parishes from Cracow, web pages of the Seniors’ Councils.

**Table 2:** Overview of age-friendly city priorities and projects in The Netherlands and Poland.

The Netherlands (The Hague)	Poland (Cracow)
**social participation**	
++/+++	++
**communication and information**	
++	+
**civic participation and employment**	
+	++
**housing**	
++	+
**transportation**	
+++	++
**community support and health services**	
++	+
++	+
+	++

+++ high (a lot of options/opportunities); ++ medium (there are some options/opportunities but not everywhere and for everyone easily accessible); + low (there are a few options/opportunities but not in a systematic way, everything depends on local policy makers, funding).
